# Prevalence of Non-Tuberculosis Mycobacterium Pulmonary Disease in HIV-1 Patients with Presumptive Pulmonary Tuberculosis in Western Kenya

**DOI:** 10.4314/ejhs.v33i5.3

**Published:** 2023-09

**Authors:** Anne Ochayo, Ronald Wamalwa, Erick Barasa, Jeremiah Zablon, George Sowayi, Tom Were, Godfrey Gitonga, Nathan Shaviya

**Affiliations:** 1 Department of Medical Laboratory Sciences, School of Public Health, Biomedical Science and Technology, Masinde Muliro University, P.O. Box 190-50100, Kakamega, Kenya; 2 National Tuberculosis Reference Laboratory & Kenyatta National Hospital, P.O Box 20723-00202, Nairobi, Kenya; 3 Department of Microbiology and Parasitology, School of Medicine, Masinde Muliro University, P.O. Box 190-50100, Kakamega Kenya

**Keywords:** non-tuberculous mycobacterium, HIV-1, Pulmonary tuberculosis

## Abstract

**Background:**

Non-tuberculous mycobacteria (NTMs) are ubiquitous, free-living, environmental saprophytic microorganisms. NTMs belong to the genus Mycobacterium which includes Mycobacterium tuberculosis (MTB). NTMs have lately been a major cause of pulmonary disease (PD) in immuno-compromised individuals including HIV-1 patients. NTMs and MTB appear similar based on microscopy, radiology, and clinical symptoms; consequently, this may lead to misdiagnosis. This study sought to establish the prevalence of NTM pulmonary disease in HIV-1 patients presumed to have pulmonary tuberculosis.

**Methods:**

A cross-sectional analytical laboratory study design was used targeting 617 adult HIV-1 infected patients presenting with presumptive pulmonary TB at Bungoma County Hospital Comprehensive Care Clinic in Western Kenya between July 2021 to June 2022.

**Results:**

A total of 75 (12.2%, 4.6 -9.8 CI) of the participants presented with presumptive MTB and had TB-like symptoms while 542 (87.8%, 12.5 -30.7 CI) were negative. Additionally, 56 (9.1%) were infected with NTMs. HIV-positive participants had a significantly higher prevalence of NTMs 62 (11.8%, 5.6 -9.2 CI) compared to 2 (2.1%, 0.4 -1.8 CI). In HIV + study participants P<0.0001. M. avium was the most prevalent NTM, 25(33.3%), followed by M. fortuitum 20 (26.7%). A significant number of the isolates were M. tuberculosis 10 (13.3%) as well as M. kansasii 8 (10.7%).

**Conclusion:**

There seems to be a high prevalence of NTMPD in HIV-1 patients which is assumed to be pulmonary TB. Differential diagnosis of the mycobacterium species is necessary to help improve disease management and outcomes in this group of patients.

## Introduction

Non-tuberculous mycobacteria (NTMs) are ubiquitous, free-living, environmental saprophytic microorganisms (1,2). These microorganisms are mostly found in natural and municipal water, soil, biofilms, aerosols, vegetation, animals, and humans(3). NTMs belong to the genus Mycobacterium which includes *M. tuberculosis* (MTB) and *M. leprae*. Studies have shown that NTMs are the genetic progenitors of *the Mycobacterium tuberculosis* Complex (MTBC) (4). Furthermore, phylogenetic analyses appear to imply that a series of gene deletions and acquisitions might have led to the evolution of MTBC into a more virulent pathogen (5,6). Approximately, 200 species of NTM have been identified, and reports from diverse countries and regions indicate that different NTMs isolated from clinical samples differ significantly by region. Nonetheless, *Mycobacterium avium* complex (MAC) seems to be the most prevalent NTM isolated clinically. Previously, NTMs have been considered to be non-pathogenic (7). Recent studies are revealing NTMs as emerging etiologic factors influencing significantly the burden of disease (8). Diseases associated with NTMs include lymphadenopathy, mycobacterial pulmonary disease, Buruli ulcer, and skin and soft tissue disease (3,9). Of these diseases, mycobacterial pulmonary disease seems to be contributing the greatest burden, especially in immune-compromised individuals (10,11).

Increasing prevalence and incidence of NTMPD globally. Asia, Western Europe, and America have had exponential growth in the previous 20 years. In the US, Donohue et al. found that NTMPD prevalence increased from 2.4 cases/100,000 in the 1980s to 15.2 in 2013 (12). Canada, the UK, Denmark, and Germany show similar trends (13–15). Similar NTMPD burdens have been detected throughout Africa. Recently, sub-Saharan Africa has a 7.5% prevalence (16). According to the research, NTMPD prevalence varies throughout African countries. Ghana (23%), Nigeria (36.0%), Uganda (9.2%), Tanzania (15.0%), Zambia (25.8%), and South Africa (30.2%) (17–22). Kenyan reports also show differing NTMPD prevalence rates. A Siaya research found 2.6% NTMPD in babies (23). These studies examined NTMPD in the general population. These studies suggest NTMPD is a neglected public health issue. However, immunocompromised people, notably HIV/AIDS patients, have a higher pulmonary disease burden. This high frequency may be owing to misclassification of NTM-related lung illness as MTB. Thus, pulmonary illness etiology is crucial for immunocompromised individuals' treatment and care.

## Materials and Methods

**Study design and population**: A cross-sectional analytical laboratory study design was used targeting adult HIV-1 infected patients presenting with presumptive pulmonary TB at Bungoma County Referral Hospital Comprehensive Care Clinic in Western Kenya between July 2021 to June 2022. Selection followed the following criteria.

**Inclusion criteria**: HIV-1 positive presenting with TB-like symptoms including chronic productive cough lasting more than 2 weeks, loss of appetite, fever, fatigue, headache, and night sweats, and consenting t participate in the study

**Exclusion criteria**: Patients on TB treatment were excluded from the study.

**HIV-1 diagnosis**: Confirmation of HIV-1 was done using rapid immunochromatographic test kit, Determine™ (Abbott Laboratories, Tokyo, Japan), and first response™ (Trinity Biotech Plc, Bray, Ireland). Per the Kenyan national HIV testing algorithm, participants were considered HIV-1 infected if they had HIV-positive results for Determine™ and HIV-1 positive results using first response™ kits.

**Smear microscopy**: Sputum samples were screened by fluorescent microscopy using Auramine O stain and smears found to be positive were confirmed by light microscopy using Ziehl-Neelsen's stain as per the standard protocols of both staining methods. Sputa were graded for positivity of AFB as per the guidelines, decontaminated according to standard guidelines, and divided into two parts.

**Line probe assay for MTB complex**: Mycobacterial DNA was extracted from one part of the decontaminated smear-positive sputum samples using GenoLyse®, VER1.0 (Hain Lifescience, GmBH, Nehren, Germany) according to the manufacturer's guidelines and stored at 4°C for a maximum of two days to batch the samples. Line Probe Assay was carried out using GenoType® MTBDRplus,VER 2.0 (Hain Lifescience, GmBH, Nehren, Germany) to look for the presence of MTB complex as well as drug resistance to rifampicin and isoniazid, as per the manufacturer's instructions.

**Culture:** Decontaminated samples of those sputum samples that were found to have no members of MTB complex were cultured on Löwenstein-Jensen (LJ) media as per the standard rotocol and incubated at 37°C for a maximum of 8 weeks. Any strain of AFB grown from these samples was put up for biochemical tests and an rRNA-based DNA hybridization assay (Accuprobe® System; Gen-Probe Inc., San Diego, CA, USA) to detect the presence of MTB complex, if any, according to the manufacturer's guidelines.

**Line probe assay for NTM**: The strains negative for MTB complex were confirmed as NTM by negative niacin accumulation test, growth on paranitrobenzoic acid (PNB) incorporated LJ media, positive catalase test, and a negative result of a ribosomal RNA-based DNA hybridization assay for *Mycobacterium tuberculosis* complex (Accuprobe® System Gen-Probe Inc., San Diego, CA, USA). DNA was extracted from these NTM using GenoLyse®, VER1.0 (Hain Lifescience, GmBH, Nehren, Germany) according to the manufacturer's instructions. Line probe assay for NTM was carried out using GenoType® *Mycobacterium* common mycobacteria (CM), VER 1.0 (Hain Lifescience, GmBH, Nehren, Germany) to identify the NTM as per the manufacturer's guidelines.

If NTM were detected in a sputum sample, a request was made to the treatment providers to organize to send three consecutive sputum samples from the patient to understand whether there was an NTM infection according to the established American Thoracic Society (ATS) criteria. Smear microscopy, culture, and LPA were then again carried out as described above.

**Ethical considerations**: Ethical approval for this study was obtained from Masinde Muliro University of Science and Technology Institutional Ethical Review Committee (Protocol: MMUST/IERC/101/2022. Permission to carry out the study was sought from the National Council of Science and Technology (NACOSTI). Written informed consent was obtained from each participant before enrolment. The participants were given a clear and understandable explanation of the procedure of the research study, including its purpose, potential risks, benefits, and alternatives. The participants had the opportunity to ask questions and ensure that all concerns have been addressed to their satisfaction. They had the right to refuse or withdraw their consent at any time. All HIV-1 infected ART-naive, TB, and NTM-infected study participants were referred for further treatment.

## Results

**Figure 1:**
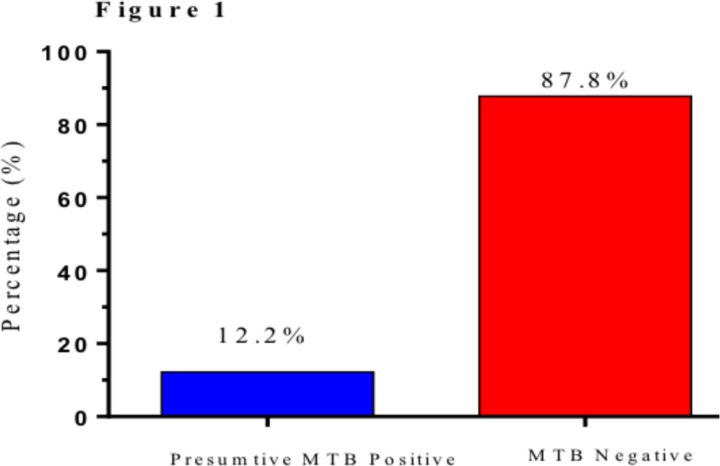
prevalence of participants with presumptive *Mycobacterium tuberculosis* (MTB) in the study site while

**Figure 2:**
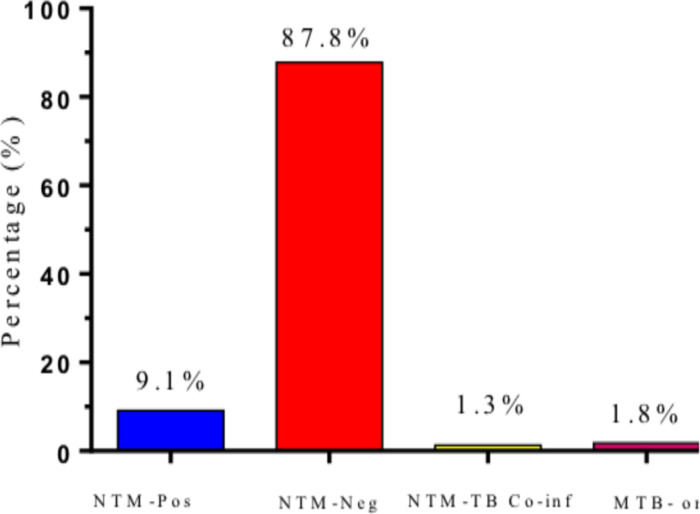
Proportions of participants with non-tuberculosis mycobacterium (NTM) after confirmatory laboratory tests

**Prevalence of NTMs**: The current study recruited 617 study participants, 523 (84.8%, 16.3 -28.6 CI) HIV-1 positive and 94 (15.2%, 6.2 -10.4 CI) supposedly “healthy” HIV-1 negative at Bungoma County referral hospital. Figure 1 below shows the overall prevalence of NTM among the study participants. A total of 75 (12.2%, 4.6 -9.8 CI) of the participants presented with presumptive MTB and had TB-like symptoms while 542 (87.8%, 12.5 -30.7 CI) were negative. In addition, 56 (9.1%) were found to have been infected with NTMs, 8 (1.3%) were NTM and MTB co-infected while 11 (1.8) were MTB infected as shown in Figure 2. Therefore, the overall prevalence of NTMs in the study participants was found to be 65 (10.5%, 5.1 -8.7 CI). NTM infection was significantly more prevalent in the HIV-1 infected participants compared to the HIV negative.

Figure 1 shows the prevalence of participants with presumptive *Mycobacterium tuberculosis* (MTB) in the study site while Figure 2 shows the proportions of participants with non-tuberculosis mycobacterium (NTM) after confirmatory laboratory tests. Non-tuberculosis mycobacterium positive (NTM-Pos), non-tuberculosis mycobacterium negative (NTM-Neg), non-tuberculosis mycobacterium and MTB co-infection (NTM-TB Co-inf) and *Mycobacterium tuberculosis* mono-infection (MTB-only).

Data on the prevalence of NTMs between the study groups is presented in Table 1 below. HIV-positive participants had a significantly higher prevalence of NTMs 62 (11.8%, 5.6 -9.2 CI) compared to 2 (2.1%, 0.4 -1.8 CI) in HIV + study participants *P*<0.0001. Moreover, 11 (2.2%, 0.3 -2.1 CI) presented with MTB in the HIV+ group while none presented with MTB in the HIV- group. The rate of NTM and MTB co-infection was also reported to be 1.5% among HIV-1 infected participants. These patients presenting with NTMs had previously been assumed to have MTB before the confirmatory laboratory tests.

**Table 1:** The prevalence of NTM infection in the study groups, HIV − (negative) and HIV+ (positive)

Variables	HIV (-), n=94	HIV (+), n=523	*P*
***Overall prevalence***			
NTM (-)	92 (97.9)	450 (86.0)	
NTM (+)	2 (2.1)	62 (11.8)	**<0.0001**
MTB (+)	0 (0.0)	11 (2.2)	
***Co-infection***			
NTM (-)	92 (97.9)	450 (86.0)	
NTM (+)	2 (2.1)	54 (10.3)	**<0.0001**
NTM (+) MTB (+)	0 (0.0)	8 (1.5)	
MTB (+)	0 (0.0)	11 (2.2)	

Non-tuberculosis mycobacterium negative (NTM -), non-tuberculosis mycobacterium positive (NTM +), non-tuberculosis mycobacterium and MTB co-infection (NTM+MTB+) and *Mycobacterium tuberculosis* mono-infection (MTB +); Data analyses for these categorical variables was done using chi-square test. *P* value <0.05 considered significant.

**Figure 3:**
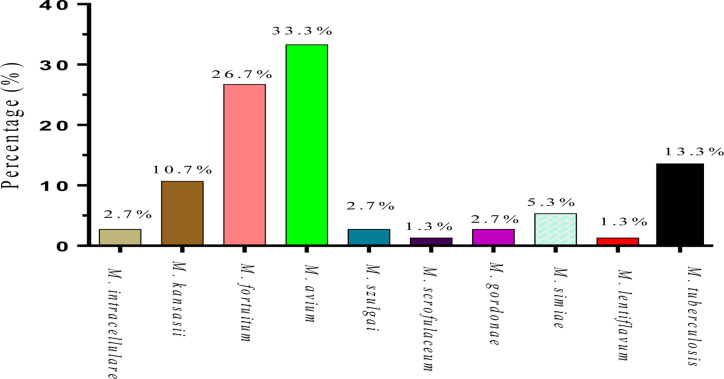
Characterized mycobacterium species from the isolates

**Characterization of mycobacterium species**: Of the 75 participants with presumptive MTB, the isolates were characterized. Figure 3 below shows the summary of the mycobacterium species characterized from the patient samples. The co-infected samples were considered to be NTMs during analysis. *Mycobacterium avium* was the most prevalent NTM, 25 (33.3%), followed by *M. fortuitum*, 20 (26.7%). A significant number of the isolates were *M. tuberculosis*, 10 (13.3%), as well as *M. kansasii*, 8 (10.7%). Other species characterized from the samples include *M. simiae*, 4 (5.3%), *M. intracellulare*, *M. gordonae*, *M. szulga*i all ,2 (2.7%), each and *M. scrofulaceum* as well as *M. lentiflavum*, 1 (1.3%) isolate each.

## Discussion

This study found 10.5% (5.1 -8.7 CI) NTM prevalence in Western Kenya. A recent Kenyan study found 19.3% prevalence in presumptive pulmonary TB patients (24). Current study focused on HIV-1-infected patients. NTMs are emerging lung disease etiological variables and sometimes misdiagnosed as pulmonary TB, especially in immunocompromised patients (25). NTMs may be mistaken as pulmonary TB, especially in immunocompromised patients. NTMs often cause pulmonary disease treated with macrolides (clarithromycin and azithromycin) plus rifampicin or rifabutin and ethambutol (26). Eliminating NTM species requires different antibiotic regimens (27).

Pulmonary TB is usually treated with isoniazid and rifampicin (28). Only 19 (25.3%, 4.5-9.6 CI) of 75 initial MTB diagnoses had MTB in this investigation. 11 (14.7%, 1.6-4.9 CI) of the 19 patients were MTB mono-infected, while 8 (10.7%, 0.8-3.8 CI) were NTM and TB co-infected. These findings have major consequences for patient outcomes because resource-limited settings do not perform confirmatory lab tests. MTB and NTMs cannot be diagnosed by radiography. It requires molecular probes to identify specific species following bacterial culture, the gold standard diagnostic test (29,30). The current study found *M. avium* to be the most prevalent NTM among HIV-1 participants with presumed MTB. Out of the 75 isolates, 25 (33.3%, 8.1- 14.8 CI) were *M. avium*.

Consistent with previous studies on *M. avium* complex (MAC), a slow-growing mycobacterium has been reported to be the most prevalent (31). This bacteria has been shown to account for the majority of cases in patients with NTMPD (32,33). Additionally, *M. fortuitum* was the second most abundant NTM at 26.7% (95%, 6.8 - 10.4 CI). Other NTMs included *M. kansasii, M. simiae, M. intracellulare*, *M. gordonae*, *M. szulgai*, *M. scrofulaceum*, and *M. lentiflavum*. Apart from MAC, *M. abscessus, M. kansasii*, and *M. xenopi* are the other NTMs whose etiology has been well characterized in NTMPD (34,35). Similar to the findings reported in a study in Iran, the results of this study revealed a wide range of NTMs in HIV-1 samples (36,37). Furthermore, previous studies have reported variations in the geographical distribution of NTMs (38,39). The current study did not report *M. xenopi* though it is important to note that the prevalence of NTMs varies geographically (31). Altogether, these findings suggest a high prevalence of NTMPD among HIV-1 patients assumed to have pulmonary TB. This points to a possibility of misdiagnoses in low-resource settings.

There seems to be a high prevalence of NTMPD in HIV-1 patients which is assumed to be pulmonary TB based on clinical presentation and radiology thought to be TB-like symptoms. Differential diagnosis is necessary to help improve disease management and outcomes in this group of patients.
